# Efficacy of Quantitative Muscle Ultrasound Using Texture-Feature Parametric Imaging in Detecting Pompe Disease in Children

**DOI:** 10.3390/e21070714

**Published:** 2019-07-22

**Authors:** Hong-Jen Chiou, Chih-Kuang Yeh, Hsuen-En Hwang, Yin-Yin Liao

**Affiliations:** 1 Division of Ultrasound and Breast Imaging, Department of Radiology, Taipei Veterans General Hospital, Taipei 11217, Taiwan; 2 School of Medicine, National Yang Ming University, Taipei 11221, Taiwan; 3 National Defense Medical Center, Taipei 11490, Taiwan; 4 Department of Biomedical Engineering and Environmental Sciences, National Tsing Hua University, Hsinchu 30013, Taiwan; 5 Department of Radiology, Taipei Veterans General Hospital, Taipei 11217, Taiwan; 6 Department of Biomedical Engineering, Hungkuang University, Taichung 43302, Taiwan

**Keywords:** Pompe disease, children, quantitative muscle ultrasound, texture-feature parametric imaging

## Abstract

Pompe disease is a hereditary neuromuscular disorder attributed to acid α-glucosidase deficiency, and accurately identifying this disease is essential. Our aim was to discriminate normal muscles from neuropathic muscles in children affected by Pompe disease using a texture-feature parametric imaging method that simultaneously considers microstructure and macrostructure. The study included 22 children aged 0.02–54 months with Pompe disease and six healthy children aged 2–12 months with normal muscles. For each subject, transverse ultrasound images of the bilateral rectus femoris and sartorius muscles were obtained. Gray-level co-occurrence matrix-based Haralick’s features were used for constructing parametric images and identifying neuropathic muscles: autocorrelation (AUT), contrast, energy (ENE), entropy (ENT), maximum probability (MAXP), variance (VAR), and cluster prominence (CPR). Stepwise regression was used in feature selection. The Fisher linear discriminant analysis was used for combination of the selected features to distinguish between normal and pathological muscles. The VAR and CPR were the optimal feature set for classifying normal and pathological rectus femoris muscles, whereas the ENE, VAR, and CPR were the optimal feature set for distinguishing between normal and pathological sartorius muscles. The two feature sets were combined to discriminate between children with and without neuropathic muscles affected by Pompe disease, achieving an accuracy of 94.6%, a specificity of 100%, a sensitivity of 93.2%, and an area under the receiver operating characteristic curve of 0.98 ± 0.02. The CPR for the rectus femoris muscles and the AUT, ENT, MAXP, and VAR for the sartorius muscles exhibited statistically significant differences in distinguishing between the infantile-onset Pompe disease and late-onset Pompe disease groups (*p* < 0.05). Texture-feature parametric imaging can be used to quantify and map tissue structures in skeletal muscles and distinguish between pathological and normal muscles in children or newborns.

## 1. Introduction

Pompe disease is a hereditary disorder that affects the neuromuscular system and is attributed to acid α-glucosidase (GAA) deficiency. The typical manifestations of the disorder involve generalized weakness of muscles in addition to cardiomyopathy, which finally end in death [1]. In general, the first muscles to be affected in this disorder are the proximal lower limb muscles as well as the paraspinal trunk muscles [2,3]. Because skeletal muscle cells exhibit glycogen granule accumulations and various degenerative changes, muscle cells are replaced by fibrous tissues and fat cells, thus disrupting the corresponding muscular architecture [4]. These processes consequently include conditions under which infants present with floppy baby syndrome. In neuromuscular disorders, some of the regularly executed diagnostic procedures are as follows: genetic analysis, muscle enzyme activity measurement, muscle biopsy procedures, and electromyography (EMG) [5,6]. 

Although EMG is often used for identifying neuromuscular diseases, its accuracy in children varies between 10% and 98% [7]. Magnetic resonance imaging (MRI) and ultrasound are among the imaging modalities that facilitate noninvasive illustration of the muscular anatomy; these modalities are consequently being increasingly integrated into neuromuscular disease diagnosis procedures [8,9,10]. Compared with MRI, ultrasound is a more child-friendly modality as it is rapid and obviates the requirement for sedation. By measuring muscle echo intensity as well as muscle thickness, ultrasound has the ability to detect muscular-disorder-induced structural changes [11,12,13,14]. Ultrasound typically represents normal muscle tissue as a low-echo-intensity structure. In contrast, muscles subjected to fat infiltration exhibit increased ultrasound beam reflections, and such reflections have a relatively bright appearance [15]. Typically, Heckmatt’s qualitative criteria based on muscle and bone echogenicities have been used for evaluating the degree of muscle abnormality in ultrasound [8,9,16]. However, these criteria have a drawback: as age increases, the echo intensity of muscles increases as well; this trend is attributable to age-related muscle replacement by fibrous tissues and fat cells [9]. Changes in system settings can result in muscles appearing as brighter structures, and such structures are likely to be misconstrued as pathological changes [9].

For detecting muscle pathology severity and identifying structural changes of muscles, quantitative muscle ultrasound can be used, which is reliable for obtaining such identification [9,12,17,18,19]. Texture analysis primarily reflects changes in a muscle’s structural echogenicity. Histograms can be used to visualize the frequency of occurrence of gray levels; accordingly, in computer programs for analyzing ultrasound images, the following statistics that constitute typical image texture parameters are extensively used for identifying abnormalities: first, second, and run-length statistics [20,21,22]. Shannon entropy has also been used as a measure of the texture information by analyzing the probability distribution for ultrasound backscattered signals [23,24,25]. Previous studies have used some linear and first-order descriptors to characterize myopathic muscles for identifying Duchenne muscular dystrophy, a disorder that is typified by homogeneously increased echogenicity levels [26,27]. The feasibility of using Shannon entropy to characterize tissues has been explored in monitoring the progress of Duchenne muscular dystrophy [28]. First-order statistics and Shannon entropy only capture the image’s non-spatial information, so they cannot fully characterize neuropathic muscles in ultrasound B-mode images [22,29]. Neuropathic processes are often associated with heterogeneous echogenicity levels in muscles that can be attributed to muscle architecture disruptions induced by the underlying pathological condition [9,13,30]. 

Molinari et al. reported higher-order statistics to be superior to first-order features in terms of classifying muscle images [22]. Gray-level co-occurrence matrix (GLCM) is a second-order statistical method of texture analysis [31]. GLCM-derived Haralick’s features have been applied to detect changes in the structures of pathological muscle tissues in ultrasound [19,22,32,33]. To enable the Shannon entropy to quantify the configurational information of an image, a GLCM has also been used to characterize the configuration of image pixels and then reflect the characterization in the computation of Shannon entropy [29,31]. We previously presented a texture-based imaging approach that involves the application of Haralick’s texture features to simultaneously preserve local and global texture information [34]. In this study, we probed the diagnostic accuracy of texture-feature parametric imaging in discriminating normal muscles from neuropathic muscles affected by Pompe disease in children. Because muscle weakness in Pompe disease is typically noticed first in the lower limbs [2,3], each child’s rectus femoris muscle and sartorius muscle were examined in this study. Seven Haralick’s texture feature parameters with various image spatial information were evaluated and used to establish corresponding parametric images. 

This paper’s remaining sections are structured as follows. In Section 2, the acquired materials and the executed methods in this study are introduced. A description of the executed clinical tests is provided in Section 3. Section 4 presents the study’s major findings and the conclusions drawn regarding the potential applications of our proposed texture-feature parametric imaging approach in muscle ultrasound. 

## 2. Materials and Methods 

### 2.1. Participants

The Institutional Review Board associated with Taipei Veterans General Hospital granted approval of the research protocol (approval number 2015-08-008B). We acquired informed consent from the legal representatives of the children examined in this study. The study included 22 patients aged 0.02–54 months with Pompe disease and 6 healthy children aged 2–12 months with normal muscles. We separated Pompe disease into two categories: infantile-onset Pompe disease (IOPD; occurring at the age of <1 year with progressive cardiac hypertrophy, hypotonia, and respiratory distress) and late-onset Pompe disease (LOPD; occurring between 1 year of age and adulthood or at the age of <1 year without cardio hypertrophy) [35]. We collected GAA mutation, activity/performance, and pathological data for the patients. The serum expression levels of the following enzymes were examined for the patients: creatine kinase (CK), alanine transaminase (ALT), lactate dehydrogenase (LDH), and aspartate transaminase (AST).

### 2.2. Ultrasound Examinations

Several ultrasound machines in our radiology department were used to perform muscle ultrasound examinations including: an Aixplorer system (Supersonic Imagine SA, Aix-en Provence, France), S2000 system (Siemens-Acuson, Mountain View, CA, USA), S3000 system (Siemens-Acuson, Mountain View, CA, USA), and LOGIQ E9 system (GE, Wauwatosa, WI, USA). These machines were equipped with linear broadband transducers operating at 5–14, 5–14, 4–9, and 4–15 MHz, respectively. The spatial resolution of these ultrasound systems ranged from 0.5 to 1 mm. Each subject was examined by the same examiner using one of these ultrasound machines. The system settings were not fixed but adjusted individually. For each subject, transverse ultrasound B-mode images of bilateral rectus femoris and sartorius muscles were obtained. For each muscle, one B-mode image was selected that included as much of the muscle as possible. Therefore, four muscle ultrasound images were measured for each subject. A doctor experienced in the analysis of muscle ultrasound images used Adobe Photoshop software (Adobe Systems, Mountain View, CA, USA) to manually outline the muscle contour, avoiding the surrounding fascia. The maximum transverse diameter of the rectus femoris and sartorius muscles in the participants ranged from 2 to 4 cm. 

### 2.3. Texture-Feature Parametric Imaging 

The GLCM is a second-order statistics method used for extracting texture features from gray-level images, which is based on information about gray levels in pairs of pixels [31]. The GLCM between gray levels *i* and *j* is defined as:

(1) $$C_{ij}|(\delta ,\theta )=\frac{P_{ij}|(\delta ,\theta )}{\sum_{i=0}^{N_{g}-1}\sum_{j=0}^{N_{g}-1}P_{ij}|(\delta ,\theta )}$$

where the matrix element *P_ij_*∣(*δ*, *θ*) represents the number of occurrences between gray levels *i* and *j,* to describe the frequency of occurrence of two pixels at a particular distance (*δ*) and angle (*θ*). The sum in the denominator represents the total number of occurrences of gray levels *i* and *j* within the window, and *N_g_* is the quantized number of gray level. The number of rows and columns in the GLCM is equal to *N_g_*. The ultrasound B-mode images were 8-bit gray-level images (256 gray levels), so we used 8 for the gray level quantization (*N_g_*) to increase the speed of computation and reduce noise [36]. The means for the columns and rows of the GLCM are, respectively, defined as:

(2) $$\mu_{x}=\sum_{i=0}^{N_{g}-1}\sum_{j=0}^{N_{g}-1}i\cdot C_{ij}$$

and

(3) $$\mu_{y}=\sum_{i=0}^{N_{g}-1}\sum_{j=0}^{N_{g}-1}j\cdot C_{ij}$$

We investigated seven texture features to quantitatively evaluate the textural characteristics of the muscles on ultrasound B-mode images. The seven texture features are defined as follows.

Autocorrelation (AUT) is used for measuring repeating patterns of gray levels in an image. A higher AUT signifies a greater amount of regularity as well as the fineness/coarseness of texture.

(4) $$AUT=\sum_{i=0}^{N_{g}-1}\sum_{j=0}^{N_{g}-1}(i\cdot j)\cdot C_{ij}$$

Contrast (CON) is used for measuring the disparity that exists between the highest and lowest values of a pixel set, with a lower CON value being typical for a block that is locally homogeneous [36].

(5) $$CON=\sum_{n=0}^{N_{g}-1}n^{2}\cdot \left\{\underset{}{\sum_{i=0}^{N_{g}-1}\sum_{j=0}^{N_{g}-1}C_{ij}|\left|i-j\right|=n}\right\}$$

Energy (ENE) measures repetitions of pairs of pixels and is dominated by the frequency of gray-level transitions to the power of two. ENE, also known as angular second moment, is a measure of the homogeneity of an image [36]. A homogeneous image results in a higher ENE value, whereas a heterogeneous region results in a lower ENE value.

(6) $$ENE=\sum_{i=0}^{N_{g}-1}\sum_{j=0}^{N_{g}-1}C_{ij}^{2}$$

Entropy (ENT), also defined as GLCM-based improved Shannon entropy, is developed to enable the Shannon entropy to quantify the spatial information of an image [29]. ENT measures the randomness of a gray-level distribution. The ENT value is expected to be high if the gray levels are distributed randomly throughout the image.

(7) $$ENT=-\sum_{i=0}^{N_{g}-1}\sum_{j=0}^{N_{g}-1}C_{ij}^{}\cdot \log\left(C_{ij}\right)$$

Maximum probability (MAXP) measures the maximum value in a pixel pair. When the occurrence of the most predominant pixel pair is high, the MAXP is high.

(8) $$MAXP=\max\left\{C_{ij}\right\}\forall (i,j)$$

Variance (VAR) measures the heterogeneity degree and is associated with the standard deviation within an image. The VAR value increases as the difference between gray-level values and the corresponding global means increases [34].

(9) $$VAR=\sum_{i=0}^{N_{g}-1}\sum_{j=0}^{N_{g}-1}{\left(i-\mu_{x}\right)}^{2}\cdot C_{ij}^{}+\sum_{i=0}^{N_{g}-1}\sum_{j=0}^{N_{g}-1}{\left(j-\mu_{y}\right)}^{2}\cdot C_{ij}^{}$$

Cluster prominence (CPR) characterizes the tendency of pixels to cluster and is a measure of asymmetry. When the CPR value is high, the image is asymmetric [36].

(10) $$CPR=\sum_{i=0}^{N_{g}-1}\sum_{j=0}^{N_{g}-1}{\left\{i+j-\mu_{x}-\mu_{y}\right\}}^{4}\cdot C_{ij}^{}$$

We chose a displacement vector of *δ* = 1 pixel in our analyses. We provided four displacement operators, which can be used to generate GLCMs along four different directions (i.e., *θ* = 0°, 45°, 90°, and 135°). A total of four GLCMs can be obtained because a GLCM can be generated along four directions. For constructing a texture-feature parametric image, we applied a 13 × 13 pixel sliding window to the ultrasound B-mode image to evaluate each local texture feature. The local texture feature was computed by averaging the four texture feature values obtained from the four GLCMs within the sliding window. Note that we selected the 13 × 13 pixel sliding window size because it is larger than the system resolution and could characterize variations in the local muscle structure [37]. When moving the sliding window throughout the ultrasound B-mode image, we used 1 pixel steps; in each movement step, we considered the new center pixel of the window as the local texture feature. This approach produced a texture-feature parametric image in the form of a map of texture feature values. The texture-feature parametric image was smaller than the ultrasound B-mode image because the pixel values at the borders in the ultrasound B-mode image were ignored. For each texture-feature parametric image, the muscle region was manually determined on the basis of the corresponding B-mode image; the relevant texture feature parameter was averaged for the entirety of the internal region of the contour.

### 2.4. Statistical Analysis

If a relatively large set of features is used for classification processes, high coefficients of correlation between two or more features necessitate the selection and integration of multiple feature attributes to improve classification performance [38]. In general, data optimality, independence, reliability, and discrimination must be included in the criteria established for the selection of significant features in classification processes [38]. Accordingly, in this study, we used the Student’s *t*-test to evaluate the level of significance of the differences between normal muscles and pathological muscles affected by Pompe disease. We assumed a derived *p*-value of <0.05 as signifying a statistically significant difference. During our comparison of *p*-values, we adjusted the level of significance by adopting the Holm–Bonferroni method. 

For feature selection, stepwise regression analysis was used to obtain the best candidate final regression model. Stepwise regression is a systematic approach to build a multilinear model by including and eliminating individual features, alternating between backward and forward [39]. The backward–forward selection begins with an initial model, and then the explanatory power of incrementally larger and smaller models is compared through F-statistics of significance. A feature to be added or removed from the set of features is chosen based on the estimated *p*-values of the F-statistics. The algorithm consists of the following steps [39]:

(1) At the beginning, the initial model is an empty model, and the entrance and exit tolerances for the *p*-values of F-statistics are 0.05 and 0.10, respectively.
(2) If any feature is not in the model and the feature has a *p*-value less than the entrance tolerance, add the feature with the smallest *p*-value to the model and repeat this step; otherwise, proceed to the next step.
(3) If any feature in the model has a *p*-value greater than the exit tolerance, remove the feature with the largest *p*-value and return to step 2; otherwise, end.

The procedure automatically stops when no feature in the model can be removed and all the next best candidates cannot be retained in the model. Then, a stable set of features is attained. Although the stepwise model has the possibility of reaching a local optimal solution, it is still widely used because of its simplicity and efficacy.

We subsequently used Fisher’s linear discriminant analysis (FLDA) to integrate selected texture feature parameters for classifying normal and pathological muscles. FLDA is a supervised classification method as it requires a class label, and is used when groups are known a priori [40]. The FLDA process involves five steps [40]:

(1) The *d*-dimensional mean vectors for the different classes from the dataset are computed.
(2) The within-class and between-class scatter matrices are calculated.
(3) The eigenvectors and corresponding eigenvalues for the scatter matrices are estimated. An eigenvalue indicates the length or magnitude of the eigenvector.
(4) The eigenvectors of the corresponding *k* largest eigenvalues are selected to form a *d* × *k* dimensional matrix *W*, where the eigenvectors are the columns of this matrix.
(5) The *W* eigenvector matrix is used to transform the original dimensional dataset into the lower dimensional dataset. This can be summarized by the matrix multiplication: *Y* = *X* × *W*, where *X* is the original *n* × *d*-dimensional dataset, and *Y* is the transformed *n* × *k*-dimensional dataset in the new subspace.

Because the selected features contained more information about our data distribution, we were interested in retaining only those eigenvectors with the highest eigenvalues to obtain the optimal feature set. The first feature set (F1) was defined as the combination of selected features for classifying the rectus femoris muscles. The second feature set (F2) was defined as the combination of selected features for classifying the sartorius muscles. The third feature set (F3) was defined as the combination of the parameters in F1 and F2. 

We used receiver operating characteristic (ROC) curve analysis to evaluate the performance of the feature sets in discriminating normal muscles from pathological muscles. Sensitivity and 1 − specificity pairs typically constitute an ROC curve, with every point along the curve representing a sensitivity/specificity pair that is related to an established decision threshold [41]. Sensitivity measures the percentage of pathological muscles that have been correctly classified. Specificity is a measure of the proportion of normal muscles that have been correctly classified. The area under the ROC curve (*Az*) could additionally be considered a potential feature.

## 3. Results

The characteristics and descriptive statistics of the Pompe disease and normal groups are listed in Table 1. In the Pompe disease group, five patients were newborns confirmed to have IOPD, and 17 patients were diagnosed as having LOPD. 

**Figure 1:**
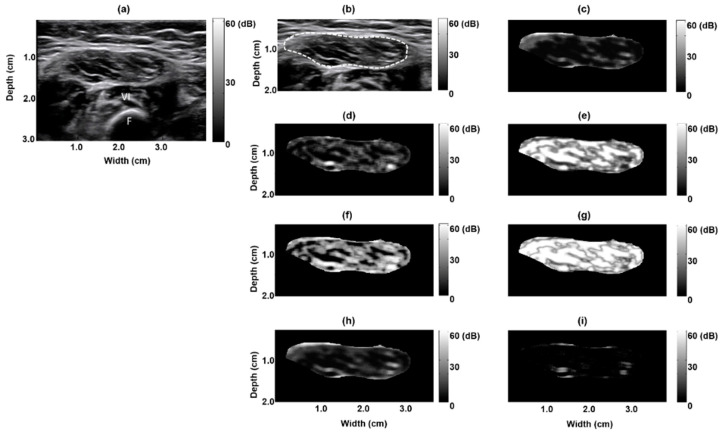
Texture-feature parametric imaging of a normal rectus femoris muscle in a 12 month old boy. (**a**) Original B-mode image, (**b**) extracted rectus femoris muscle region (indicated by the white dashed line) in the B-mode image, (**c**) autocorrelation image, (**d**) contrast image, (**e**) energy image, (**f**) entropy image, (**g**) maximum probability image, (**h**) variance image, and (**i**) cluster prominence image. F: femur bone reflection, VI: vastus intermedius muscle.

**Figure 2:**
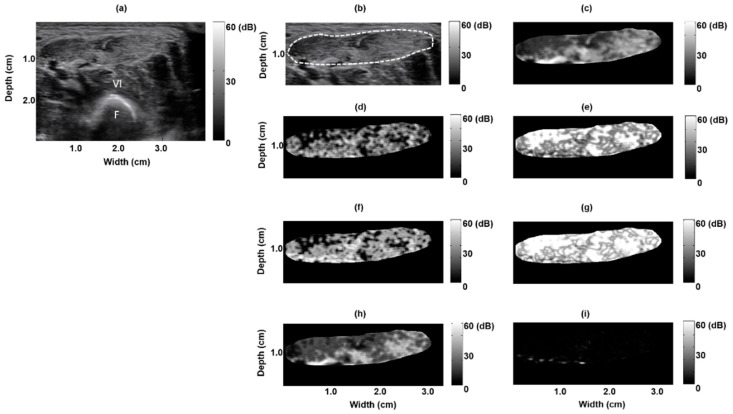
Texture-feature parametric imaging of a pathological rectus femoris muscle in a 10 day old boy with infantile-onset Pompe disease. (**a**) Original B-mode image, (**b**) extracted rectus femoris muscle region (indicated by the white dashed line) in the B-mode image, (**c**) autocorrelation image, (**d**) contrast image, (**e**) energy image, (**f**) entropy image, (**g**) maximum probability image, (**h**) variance image, and (**i**) cluster prominence image.

**Figure 3:**
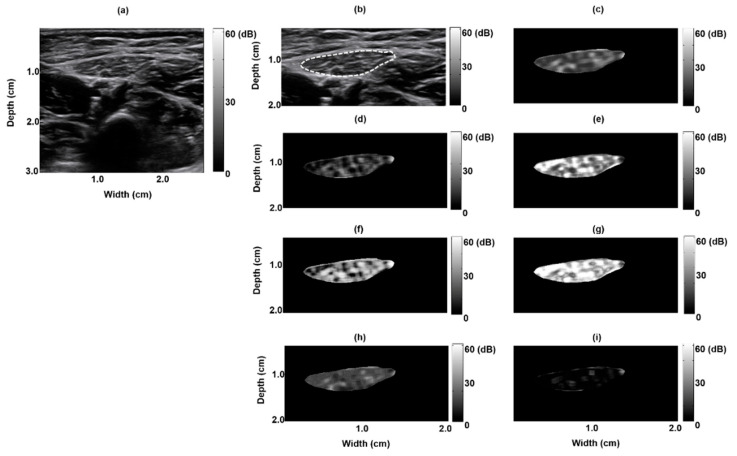
Texture-feature parametric imaging of a normal sartorius muscle in a 12 month old boy. (**a**) Original B-mode image, (**b**) extracted sartorius muscle region (indicated by the white dashed line) in the B-mode image, (**c**) autocorrelation image, (**d**) contrast image, (**e**) energy image, (**f**) entropy image, (**g**) maximum probability image, (**h**) variance image, and (**i**) cluster prominence image.

**Figure 4:**
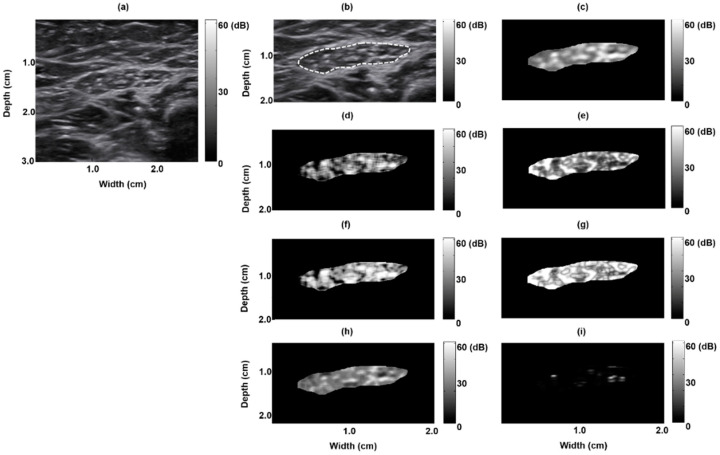
Texture-feature parametric imaging of a pathological sartorius muscle in a five month old boy with late-onset Pompe disease. (**a**) Original B-mode image, (**b**) extracted sartorius muscle region (indicated by the white dashed line) in the B-mode image, (**c**) autocorrelation image, (**d**) contrast image, (**e**) energy image, (**f**) entropy image, (**g**) maximum probability image, (**h**) variance image, and (**i**) cluster prominence image.

Figure 1a depicts a B-mode image of a normal rectus femoris muscle: clear borders with low echo intensity. The muscle region, delineated by the dashed white line in Figure 1, was extracted to form a texture-feature parametric image (Figure 1b); seven parametric images based on the seven texture features were created (Figure 1c–i). Figure 2 depicts the B-mode image of a pathological rectus femoris muscle (i.e., the muscle of a patient with Pompe disease); the image has blurry borders and increased internal echoes (Figure 2a,b). On the basis of this image, we derived seven texture-feature parametric images (Figure 2c–i). We compared a normal sartorius muscle with the sartorius muscle of a patient with Pompe disease and found that the pathological sartorius muscle exhibited a higher echo intensity level (Figure 3a and Figure 4a). Figure 3b and Figure 4b present images depicting the muscle boundaries of the normal sartorius muscle and the sartorius muscle of the patient with Pompe disease, respectively, and Figure 3c–i and Figure 4c–i depict the corresponding texture-feature parametric images. The images displayed in all figures were formed with a dynamic range of 60 dB and composed of shades of gray, varying from black at the weakest intensity to white at the strongest. The results show that the shading in the AUT, CON, ENE, ENT, MAXP, and VAR images differed between the normal and pathological muscles, with a greater amount of white shading for the pathological muscle than for the normal muscle. The intensity in the CPR image was lower for pathological muscle than for normal muscle.

**Figure 5:**
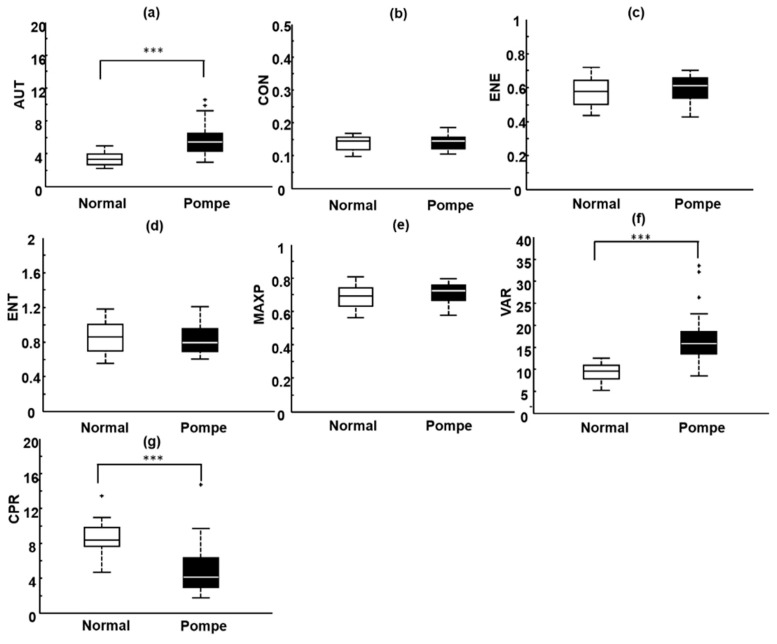
Box plots of the distributions of the seven parameters for normal rectus femoris muscles and pathological rectus femoris muscles affected by Pompe disease. (**a**) AUT: autocorrelation; (**b**) CON: contrast; (**c**) ENE: energy; (**d**) ENT: entropy; (**e**) MAXP: maximum probability; (**f**) VAR: variance; (**g**) CPR: cluster prominence; *** *p* < 0.001.

Box plots were used to represent the CON, AUT, ENE, ENT, MAXP, CPR, and VAR distributions for normal and pathological rectus femoris muscles (Figure 5), providing a quantitative description of all texture feature parameters. We found that the AUT, VAR, and CPR estimates were appropriate for distinguishing normal rectus femoris muscles from pathological rectus femoris muscles. For normal and pathological rectus femoris muscles, the average AUT, VAR, and CPR estimates were 3.91 ± 1.13 and 5.62 ± 1.78 (*p* = 0.0004), 9.17 ± 2.30 and 15.60 ± 5.51 (*p* < 0.0001), and 8.12 ± 2.44 and 4.06 ± 2.53 (*p* < 0.0001), respectively. However, the average CON, ENE, ENT, and MAXP estimates for normal and pathological rectus femoris muscles were associated with *p*-values >0.05.

**Figure 6:**
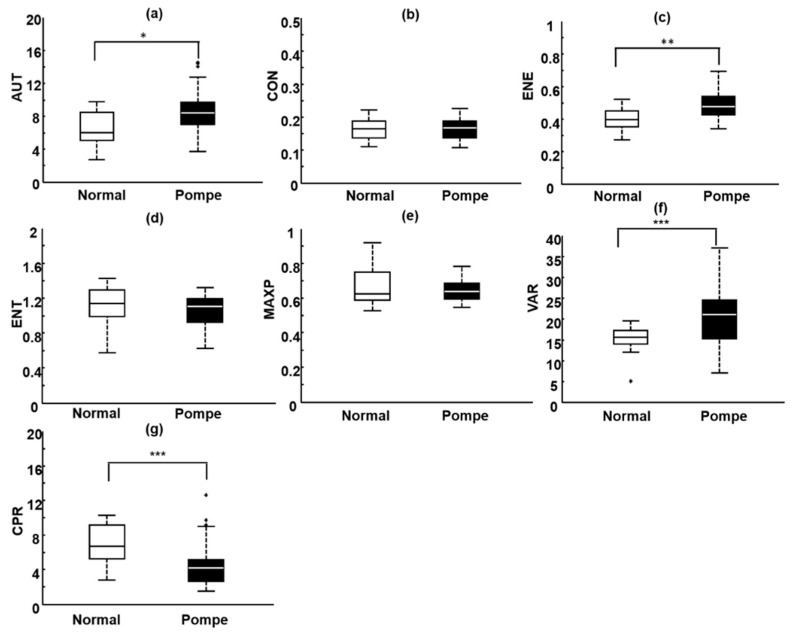
Box plots of the distributions of the seven parameters for normal sartorius muscles and pathological sartorius muscles affected by Pompe disease. (**a**) AUT: autocorrelation; (**b**) CON: contrast; (**c**) ENE: energy; (**d**) ENT: entropy; (**e**) MAXP: maximum probability; (**f**) VAR: variance; (**g**) CPR: cluster prominence; * *p* < 0.05; ** *p* < 0.01; and *** *p* < 0.001.

Box plots were also used to represent the CON, AUT, ENE, ENT, MAXP, CPR, and VAR distributions for normal and pathological sartorius muscles (Figure 6). The AUT, ENE, VAR, and CPR estimates exhibited statistically significant differences and thus could be used for distinguishing normal sartorius muscles from pathological sartorius muscles. In contrast, the CON, ENT, and MAXP estimates did not differ significantly. For normal and pathological sartorius muscles, the average AUT, ENE, VAR, and CPR estimates were 6.00 ± 2.18 and 8.01 ± 2.64 (*p* = 0.0133), 0.40 ± 0.07 and 0.48 ± 0.08 (*p* = 0.0011), 15.04 ± 3.84 and 21.62 ± 7.64 (*p* = 0.0002), and 6.55 ± 2.29 and 4.25 ± 2.46 (*p* = 0.0071), respectively.

**Figure 7:**
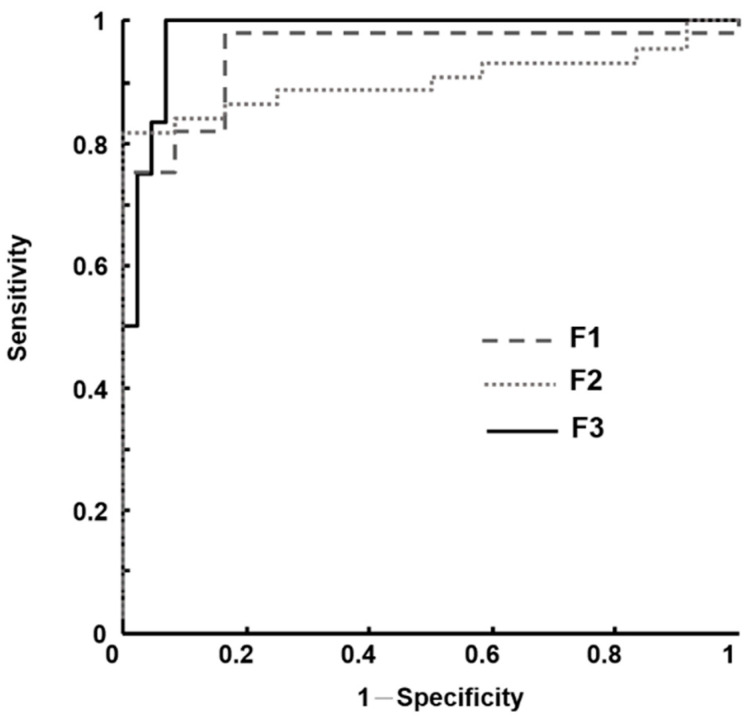
Receiver operating characteristic (ROC) curves of each feature set. F1: comprising the variance and cluster prominence for rectus femoris muscles. F2: comprising the energy, variance, and cluster prominence for sartorius muscles. F3: constituting a combination of F1 and F2.

In stepwise regression, we selected VAR and CPR as the optimal feature set for classifying normal and pathological rectus femoris muscles, whereas ENE, VAR, and CPR were selected for the optimal feature set to distinguish between normal and pathological sartorius muscles. The FLDA was used for searching for a linear combination of the selected features that best distinguished between normal and pathological muscles. VAR and CPR for the rectus femoris muscles constituted F1; ENE, VAR, and CPR for the sartorius muscles constituted F2; and a combination of the parameters in F1 and F2 constituted F3. The classification performances of these feature sets were evaluated using ROC analysis. We found F3 produced the best performance (Figure 7 and Table 2), with the highest *Az* (0.98 ± 0.02) and 100% specificity, whereas F1 and F2 produced 83.3% and 91.7% specificity, respectively.

We observed that the CPR estimates for rectus femoris muscles and the AUT, ENT, MAXP, and VAR estimates for sartorius muscles were different between the IOPD and LOPD groups (Table 3). These parameters were associated with *p*-values <0.05 for the IOPD and LOPD groups.

## 4. Discussion

Muscle ultrasound is a beneficial method for diagnosing patients with suspected muscle diseases or neuromuscular disorders. Ultrasound changes observed in diseased muscles include increased echogenicity within muscle substance, atrophic change in muscles, and loss of bone echo. Several studies have demonstrated that qualitative and quantitative ultrasound methods can be used to assess the presence and degree of muscle pathology [12,13,14,15,16,17,18,19]. Muscles that are determined to be normal exhibit a relatively hypoechoic appearance; however, on ultrasound images, different muscles exhibit distinct appearances (distinct normal ranges of echo intensity), and this is attributed to different fibrous tissue proportions and muscle fiber orientations [9]. Many conditions affect muscle ultrasound signal intensity, such as differences in patient age, system settings, and imaging modality. Although qualitative rating scales can be applied in ultrasound systems, they are subjective as they depend on the examiner’s expertise [8,9,16]. For these reasons, an adequate quantitative ultrasound method for evaluating neuromuscular disorders must be able to describe changes in muscle microstructures during fatty infiltration and be independent of system settings. Studies have confirmed that texture-feature parametric imaging can be a useful approach for characterizing breast masses or fatty livers [21,34]. Microstructure and macrostructure echo information is considered simultaneously in this approach to minimize texture analysis errors due to artifact interference. Texture-feature parametric imaging achieves image dynamic range consistency by applying normalization processes, thus overcoming dependence on system settings. 

In this study that included children with normal muscles and those with muscles affected by Pompe disease, we used B-mode ultrasound. The resulting B-mode images depicted clearly visible boundaries of normal muscles. This visibility is attributable to the highly reflective nature of the epimysia. Normal sartorius muscles were found to be generally more homogeneously hyperechoic than normal rectus femoris muscles. Consequently, we characterized the sartorius muscles and rectus femoris muscles in the Pompe disease and normal groups of children separately. The rectus femoris muscles and sartorius muscles in the children with Pompe disease exhibited increased echogenicity. Scholten et al. reported that muscle echo intensity levels increase with age in adults; in contrast, age was found to have no effect on muscle echo intensity in children [4]. Accordingly, connective tissue and fat infiltration could be the most likely explanation for the observed augmentation of muscle echo intensity in children. We noted that for both the rectus femoris muscles and sartorius muscles, the AUT, VAR, and CPR estimates exhibited statistically significant differences in distinguishing normal muscles from pathological muscles. Compared with normal muscles, pathological muscles had a higher AUT, reflecting a higher degree of fineness/coarseness; a higher VAR, representing a higher degree of heterogeneity; and a lower CPR, demonstrating a higher degree of symmetry.

The optimal feature sets were obtained using stepwise regression and FLDA. F1 (i.e., comprising VAR and CPR for rectus femoris muscles) yielded high sensitivity, which can improve the diagnosis of rectus femoris muscles affected by Pompe disease. This feature set exhibited weak specificity (less than 85.0%), which can influence the identification of normal rectus femoris muscles. We additionally noted a similar phenomenon when F2 (i.e., comprising ENE, VAR, and CPR for sartorius muscles) was used to classify normal and pathological sartorius muscles. F2 had low sensitivity (84.1%) because some pathological sartorius muscles resembled normal muscles in terms of echogenicity. A possible reason for this finding is that normal sartorius muscles exhibit a similar structure: they are divided by hyperechoic transverse tendinous inscriptions into segments. We subsequently combined F1 and F2 into F3 to improve the detection of Pompe disease, achieving a specificity of 100% and a sensitivity of 93.2%. This implies that the optimal texture feature parameter sets for rectus femoris and sartorius muscles are independent and complementary; therefore, ensuring their appropriate combination can enhance Pompe disease classification.

We found that some texture feature parameters for rectus femoris muscles and sartorius muscles were significantly different between the IOPD and LOPD groups. This result is consistent with the findings of Hwang et al., who used a muscle ultrasound scoring system based on modified Heckmatt’s qualitative criteria to distinguish IOPD from LOPD, achieving 100.0% sensitivity and 84.0% specificity [42]. They proposed that the echogenicity of muscle tissues in newborns and infants can increase because newborns and infants have small muscle fibers and a relatively high proportion of endomysial and perimysial connective tissues. Their findings revealed that the muscle ultrasound score is correlated with the serum levels of laboratory parameters in the diagnosis of IOPD. However, the qualitative scores obtained from subjective assessments can vary dramatically and affect the reliability of the results. Therefore, we suggest that disease severity can be estimated using changes in the texture feature parameters of muscles in patients with IOPD. Although a fluorometric GAA activity assay based on dried blood spots is the predominant method for diagnosing Pompe disease, it does not effectively distinguish between IOPD and LOPD or false-positive cases with pseudodeficiency mutation [42,43]. In future research, texture-feature parametric imaging will be a useful method for differentiating IOPD from LOPD and as a correlate of changes in clinical parameters.

Although this study offers valuable insight into Pompe disease identification using quantitative muscle ultrasound, it has some limitations. The first limitation is the small sample size; the sample must be increased to improve the effectiveness of identifying Pompe disease severity. Second, although all texture-feature parametric images have the same dynamic range to ensure consistency among ultrasound machines, further research on standardization approaches among scanning protocols and ensuring the reproducibility of measured values is warranted. The sliding window size used for constructing texture-feature parametric images should be dependent on different ultrasound equipment and the different ages of subjects. Third, inter- and intra-reader agreement regarding the texture feature parameters of rectus femoris and sartorius muscles should be considered during data collection.

In conclusion, our study demonstrated that texture-feature parametric imaging can be used to quantify and map tissue structures in skeletal muscles and to differentiate pathological from normal muscles in children. Such imaging is therefore a potentially useful diagnostic tool for IOPD.

## Figures and Tables

**Table 1:** Characteristics of participants.

Variable	Normal (*n* = 6)	Pompe Disease (*n* = 22)	
		LOPD	IOPD
**Male:Female**	4:2	12:5	2:3
**Age (Mean)**	12.14 months	21.82 months	0.04 months
**GAA activity by DBS (Mean ± SD)**	-	0.40 ± 0.22 μm/L/h	0.08 ± 0.03 μm/L/h
**LDH level (Mean ± SD)**	-	459.1 ± 271.3 U/L	511.2 ± 90.6 U/L
**CK level (Mean ± SD)**	-	314.7 ± 329.7 U/L	661.0 ± 384.9 U/L
**ALT level (Mean ± SD)**	-	51.7 ± 50.4 U/L	41.4 ± 18.1 U/L
**AST level (Mean ± SD)**	-	90.8 ± 88.2 U/L	94.4 ± 17.8 U/L

Note: ALT: alanine transferase; AST: aspartate transferase; CK: creatine kinase; DBS: dried blood spot; GAA: glucosidase alpha acid; IOPD: infantile-onset Pompe disease; LDH: lactate dehydrogenase; LOPD: late-onset Pompe disease; U/L: units per liter.

**Table 2:** Individual performance assessed by the area under receiver operating characteristic curve (*Az*) values (mean ± standard error and 95% confidence intervals), accuracy, specificity, sensitivity, positive predictive value (PPV), and negative predictive value (NPV) of each feature set in discriminating between children with and without neuropathic muscles for Pompe disease.

Feature Sets Performance	F1 *	F2	F3
Accuracy (%)	94.6	85.7	94.6
Specificity (%)	83.3	91.7	100
Sensitivity (%)	97.7	84.1	93.2
PPV (%)	95.6	97.6	85.7
NPV (%)	90.9	78.6	100
*Az* (mean ± standard error)	0.95 ± 0.03	0.90 ± 0.04	0.98 ± 0.02
*Az* (95% CI)	0.88–1.00	0.82–0.98	0.95–1.00

* F1: comprising the variance and cluster prominence for rectus femoris muscles. F2: comprising the energy, variance, and cluster prominence for sartorius muscles. F3: constituting a combination of F1 and F2.

**Table 3:** Mean, SD, and *p*-values derived from Student’s *t*-test of significant texture feature parameters for the infantile-onset Pompe disease (IOPD) group and the late-onset Pompe disease (LOPD) group.

Texture Feature Parameters	IOPD	LOPD	
	Mean ± SD		*p*-Value
CPR for rectus femoris muscles *	6.00 ± 2.18	8.01 ± 2.64	<0.0001
AUT for sartorius muscles	5.90 ± 1.49	8.63 ± 2.60	0.0002
ENT for sartorius muscles	0.89 ± 0.16	1.05 ± 0.17	0.0151
MAXP for sartorius muscles	0.69 ± 0.07	0.62 ± 0.06	0.0176
VAR for sartorius muscles	16.52 ± 4.07	23.12 ± 7.84	0.0071

*AUT: autocorrelation; CPR: cluster prominence; ENT: entropy; MAXP: maximum probability; VAR: variance.
